# Augmenting Intrinsic Fenton-Like Activities of MOF-Derived Catalysts via *N*-Molecule-Assisted Self-catalyzed Carbonization

**DOI:** 10.1007/s40820-019-0319-4

**Published:** 2019-10-17

**Authors:** Chengdong Yang, Mi Zhou, Chao He, Yun Gao, Shuang Li, Xin Fan, Yi Lin, Fei Cheng, Puxin Zhu, Chong Cheng

**Affiliations:** 1 Textile Institute, College of Biomass Science and Engineering, Sichuan University, Chengdu, 610065 People’s Republic of China; 2 College of Polymer Science and Engineering, State Key Laboratory of Polymer Materials Engineering, Sichuan University, Chengdu, 610065 People’s Republic of China; 3 Functional Materials, Department of Chemistry, Technische Universität Berlin, Hardenbergstraße 40, 10623 Berlin, Germany; 4 Department of Chemistry and Biochemistry, Freie Universität Berlin, Takustrasse 3, 14195 Berlin, Germany

**Keywords:** Carbon catalysts, Metal–organic-framework, Self-catalytic carbonization, Fenton-like reactions, Organic pollutions

## Abstract

To overcome the ever-growing organic pollutions in the water system, abundant efforts have been dedicated to fabricating efficient Fenton-like carbon catalysts. However, the rational design of carbon catalysts with high intrinsic activity remains a long-term goal. Herein, we report a new *N*-molecule-assisted self-catalytic carbonization process in augmenting the intrinsic Fenton-like activity of metal–organic-framework-derived carbon hybrids. During carbonization, the *N*-molecules provide alkane/ammonia gases and the formed iron nanocrystals act as the in situ catalysts, which result in the elaborated formation of carbon nanotubes (in situ chemical vapor deposition from alkane/iron catalysts) and micro-/meso-porous structures (ammonia gas etching). The obtained catalysts exhibited with abundant Fe/Fe–N_*x*_/pyridinic-N active species, micro-/meso-porous structures, and conductive carbon nanotubes. Consequently, the catalysts exhibit high efficiency toward the degradation of different organic pollutions, such as bisphenol A, methylene blue, and tetracycline. This study not only creates a new pathway for achieving highly active Fenton-like carbon catalysts but also takes a step toward the customized production of advanced carbon hybrids for diverse energy and environmental applications.

**Electronic supplementary material:**

The online version of this article (10.1007/s40820-019-0319-4) contains supplementary material, which is available to authorized users.

## Highlights

- Metal–organic-framework-derived carbon hybrids with elaborated nanostructures were prepared via *N*-molecule- assisted self-catalytic carbonization process.
- The enriched Fe/Fe-N_*x*_/pyridinic-N active species, porous structures, and conductive carbon nanotubes can synergically augment the intrinsic catalytic activities.

**Electronic supplementary material:**

The online version of this article (10.1007/s40820-019-0319-4) contains supplementary material, which is available to authorized users.

## Graphical abstract

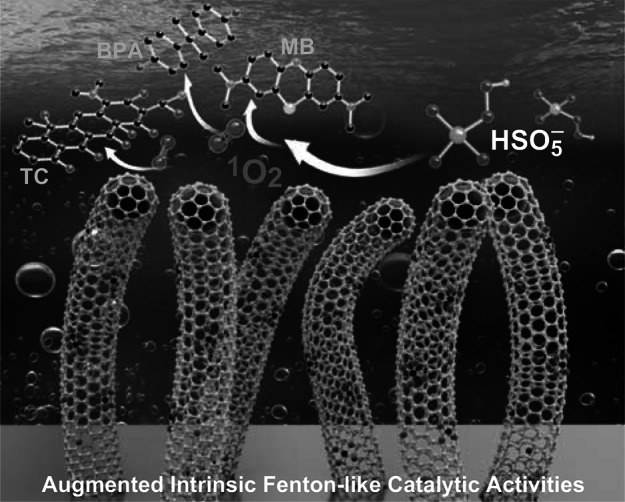

## Introduction

Water contaminations by diverse kinds of organic pollutants, such as the endocrine disruptors from plastic manufacture, dyeing wastewaters, and antibiotic used in cultivation industry, have led to severe environmental and human health problems [1–7]. In recent years, peroxymonosulfate (PMS)-based Fenton-like reactions have shown great potential to be utilized to generate reactive radicals for the degradation of organic pollutants in wastewater [8, 9]. Unfortunately, high concentrations of PMS were required in aqueous environments to generate enough radicals to degrade organic molecules. To overcome these issues, intensively studies have been focused on developing high-efficient methodologies toward the fabrication of advanced materials for PMS-based Fenton-like reactions [10–12]. Recently, various transition metal-based materials have been investigated as catalysts to activate PMS for the efficient degradation of organic pollutants, especially the nanostructured carbon hybrids with excellent conductivity, competitive catalytic activity, and prominent synergic effects [13–15]. However, the rationally designed carbon hybrids with elaborate nanostructures, high density of active centers, and facile fabrication processes are still rarely reported for the PMS-based catalytic degradations [16, 17].

Metal–organic-framework (MOFs), constructed from metal nodes/clusters and polydentate organic linkers, have attracted enormous attention [18–21]. Owing to their varied structural features, such as high surface area, tunable metal ions/organic linkers, and facile structural controllability, MOFs have been used as suitable starting materials for fabricating heteroatoms or metal particle-doped carbon hybrids, which have been applied in the fields of electro/photocatalysis, batteries, nanomedicines, etc. [22–25]. For instance, we have reported using MOF coating to achieve highly active atomic Fe–N_*x*_ catalytic centers on meso-porous carbon nanofibers for advanced oxygen electrode in metal-air batteries [26]. In another work, MOF nanoparticles have been applied as a precursor to fabricate the Fe/Fe_3_C@N-doped porous carbon catalyst for Fenton-like reactions [27]. However, many of the currently reported MOFs-derived carbon catalysts suffered from the low surface area, insufficient metal-N active centers, and poor balance of active defects and graphitization degree [28, 29]. Recently, in situ growth of carbon nanotubes (CNTs) by the pyrolysis of MOFs with reductive gas has been reported with potential benefits [30, 31], which ensures the high surface area and inhibits the aggregation of metal particles [32–35].

Herein, to augment the intrinsic catalytic activity toward the degradation of organic pollutants in wastewater, for the first time, we report a new *N*-molecule-assisted self-catalytic carbonization process in designing the MOFs-derived Fenton-like catalysts. During the carbonization, the *N*-molecules provide alkane/ammonia gases and the formed iron nanocrystals act as the in situ catalysts, which result in the elaborated formation of conductive carbon nanotubes and micro-/meso-porous structures via a combined process of in situ chemical vapor deposition (alkane/iron catalysts) and ammonia gas etching. Notably, this unique carbonization process significantly enhances its intrinsic Fenton-like activities due to the synergic effects of the enriched Fe/Fe–N_*x*_/pyridinic-N active species, micro-/meso-porous structures, and conductive carbon nanotubes. Consequently, these carbon hybrids exhibit high removal efficiency of endocrine disruptor (bisphenol A, BPA), industrial dye (methylene blue, MB), and widely used antibiotic in cultivation industry (tetracycline, TC). This study provides a facile and controllable method for the rational design of carbon hybrids with elaborated nanostructures and increased catalytic active sites, thus exhibiting promising potential in Fenton-like catalysis and many other related catalytic/electrochemical reactions.

## Experimental Section

### Materials

Fe(NO_3_)_3_·9H_2_O, 2-Aminoterephthalic, peroxymonosulfate (PMS), 5,5-dimethyl-1-pyrroline-N-oxide (DMPO), methylene blue (MB), bisphenol A (BPA), and tetracycline (TC) were purchased from Aladdin Co., China. Dimethyl Formamide (DMF), ethyl alcohol, NaOH, and dicyandiamide (DCDA) were obtained from the Kelong chemical reagent factory, China. All reagents were used without further purification.

### Synthesis of Fe-Based MIL-88B-NH_2_ Nanostructures

MIL-88B-NH_2_ was prepared according to the previous reports with minor modification [36]. In a typical synthesis, Fe (NO_3_)_3_·9H_2_O (0.399 g, 0.99 mmol) and 2-aminoterephthalic (0.179 g, 0.99 mmol) were dissolved in 10 mL DMF in a sealed reactor until total dissolution. Then, 0.2 mL NaOH (0.5 M) was added dropwise. After thoroughly mixed, the sealed reactor was transferred to an oil bath and heated at 110 °C for 7 h to obtain the MIL-88B-NH_2_. Finally, the products were washed by DMF and ethyl alcohol for three times and dried at 50 °C for overnight.

### Fabrication of MIL/CNT–Fe Catalysts

To achieve the *N*-molecule-assisted self-catalyzed carbonization processes, the as-prepared MIL-88B-NH_2_ (50 mg) was placed at one end of the crucible and *N*-molecule (DCDA, 250 mg) was placed at another end of the crucible. Then, the crucible was covered and transferred to a nitrogen oven and heated to 800 °C with a ramp rate of 2 °C min^−1^ and hold for 2 h to yield the MIL/CNT–Fe-800. We have optimized the mass ratios of MIL-88B-NH_2_ and DCDA from 1:3 to 1:8 to yield the MIL/CNT–Fe-3/8, and the results show that the MIL/CNT–Fe-800 at 1:5 shows the optimized morphology and catalytic performance. Therefore, in this study, we will focus on the product of MIL/CNT–Fe-800. If no special declaration, all the MIL/CNT–Fe-800 discussed in this paper is referred to the products prepared from 1:5 (MIL-88B-NH_2_ and DCDA ratio). In order to study the influence of different carbonization temperatures, the precursors with the mass ratio of MIL-88B-NH_2_/DCDA at 1:5 have also been carbonized at 700 and 900 °C at the same condition, and the products were coded as MIL/CNT–Fe-700 and MIL/CNT–Fe-900. In a controlled experiment, the MIL-88B-NH_2_ precursor was carbonized directly at 800 °C with a ramp rate of 2 °C min^−1^ for 2 h without adding DCDA, and this product was coded as MIL-Fe-800.

### Measurements of Fenton-Like Catalytic Activities

The catalytic performances of different catalysts were conducted via activation of PMS (0.1 g L^−1^) for removal of BPA, MB, and TC (20 mg L^−1^) with initial pH = 6 at room temperature [8]. Notably, the pH value of TC solution was adjusted by either 0.1 M NaOH or 0.1 M HCl aqueous solution. To reveal the activity of Fe nanoparticles, the catalysts are oxidized to transfer the Fe nanoparticles into the Fe_2_O_3_, and the products are coded as O-MIL/CNT–Fe-800 and O-MIL-Fe-800, respectively, by heating at 350 °C for 4 h with a ramp rate at 2 °C min^−1^ according to a previous report [20]. After that, the Fenton-like catalytic activities of these oxidized catalysts are compared with the pristine catalysts at the same condition.

In all the experiments, 5 mg catalyst was added into 50 mL pollutants solution and stirred for 15 min to get the absorption and desorption equilibrium. Then, 10 mg PMS was added into the solution to trigger the Fenton-like reaction. Besides, at each reaction interval, 2 mL solution was taken out and the reaction was immediately quenched with 2 mL methyl alcohol. What is more, in each recyclability experiment, the catalyst was collected by the magnet and followed with a anneal treat. The concentration of pollutions was evaluated by UV spectrum. The reference of the spectrum was collected by deionized water. The standard curve of each pollutant was obtained by detecting pollutants with the specific concentration, according to the Lambert–Beer’s law: *A *= *Kbc* where *A* is absorbance; *K* is molar absorption coefficient. *b* is the thickness of the absorbing layer. *c* is the concentration of pollution. Finally, the reaction rate was evaluated by pseudo-first-order equation, shown as follows: ln (*C*_0_/*C*) = *kt*, where *C*_0_ is the initial concentration of pollutant. *k* is the apparent constant rate, *t* was the reaction time [7, 37].

## Results and Discussion

### Structural and Morphological Characterizations

**Fig. 1:**
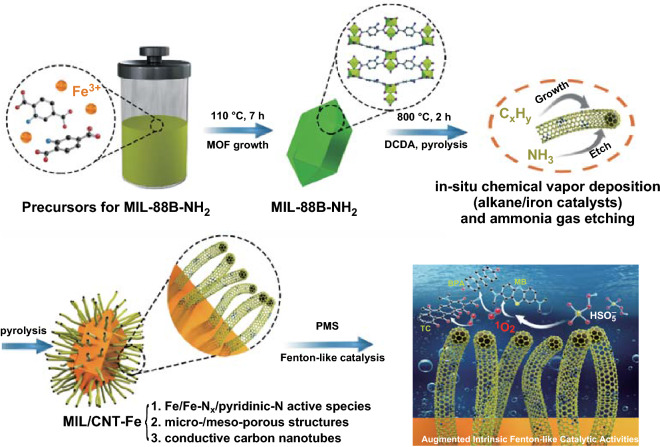
Illustrated formation procedures for the iron(III)-2-aminoterephthalic frameworks (MIL-88B-NH_2_, MIL = Materials from the Lavoisier Institute) and the resulted CNTs–Fe-decorated carbon hybrids (MIL/CNT–Fe), and the corresponding schematic image of Fenton-like reactions toward the organic pollutants

**Fig. 2:**
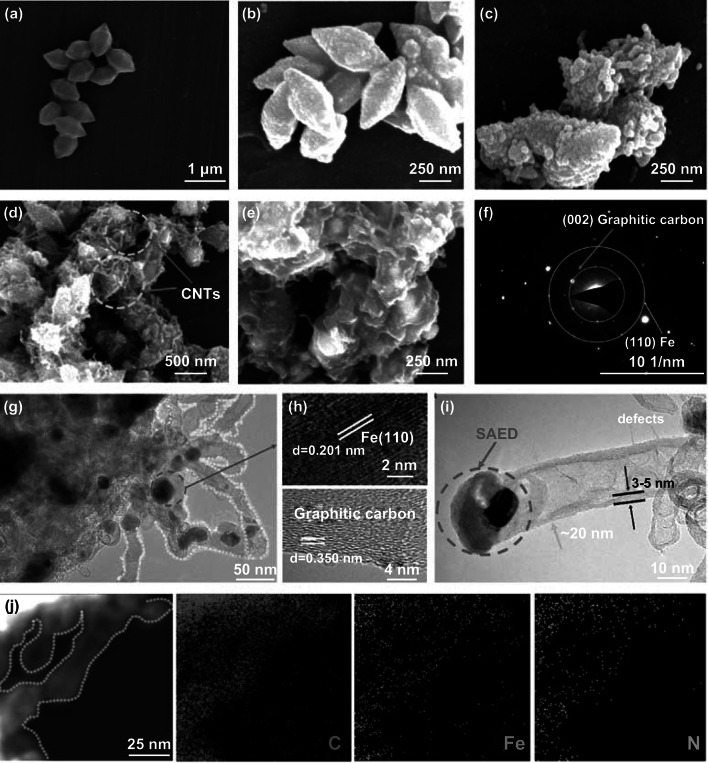
SEM images of **a** MIL-88B-NH_2_, **b** MIL-Fe-800, **c** MIL/CNT–Fe-700, **d** MIL/CNT–Fe-800, and **e** MIL/CNT–Fe-900. **f** SAED patterns for MIL/CNT–Fe-800 (marked in **i** by red circle and stated by “SAED”). **g** TEM images of MIL/CNT–Fe-800. **h** HRTEM images of MIL/CNT–Fe-800. **i** TEM image of single CNT–Fe structure for MIL/CNT–Fe-800. **j** EDX mapping of MIL/CNT–Fe-800

The schematic preparation procedures of the MIL-88B-NH_2_ (Figs. S1, S2) and MIL/CNT–Fe are illustrated in Fig. 1. In brief, we employed a Fe-based MOF, MIL-88B-NH_2_, as a model MOF precursor. Figures 2a and S2 show the typical morphology of spindle-like MIL-88B-NH_2_ with uniform size (400–500 nm wide and 800–900 nm long, aspect ratio = 1.60–2.25). The powder X-ray diffraction (PXRD) pattern (Fig. S3) of MIL-88B-NH_2_ demonstrates the formation of homogeneous phase materials with high crystallinity [27]. As shown in Fig. 2b, the carbonization of MIL-88B-NH_2_ without adding DCDA is conducted as the control, the obtained MIL-Fe-800 exhibits maintained spindle-like structures as the pristine MOF, and there is no growth of CNTs on the surface.

For the self-catalyzed carbonization process, the dicyandiamide (DCDA) is used as a model *N*-molecule, which can provide alkane/ammonia gases for the formation of CNTs and micro-/meso-porous structures via a combined process of chemical vapor deposition (alkane/iron catalysts) and ammonia gas etching. The iron catalysts are derived from the in situ formed iron nanocrystals during the carbonization process of Fe-based MOF [10, 38]. As shown in Fig. 2c–e, after adding DCDA at the mass ratio of 1:5, the yielded MIL/CNT–Fe-800 shows abundant CNT nanostructures on the spindle-like particles. While, for the particles obtained from 700 or 900 °C, as shown in Fig. 2c, e, there is almost no apparent CNT formation on the products, and only the MIL/CNT–Fe-900 shows a few amounts of CNTs. Furthermore, the influence of DCDA contents in elaborating the CNT nanostructures during self-catalyzed carbonization has also been investigated by changing the mass ratios of MIL-88B-NH_2_: DCDA from 1:3, 1:5, to 1:8. It has been observed that when the mass ratio is set as 1:5, the optimal hierarchical structures with abundant CNTs (by scan electron microscope (SEM) as shown in Figs. S4, S5) and catalytic performances (will be discussed in Sect. 3.2) of the MIL/CNT–Fe can be obtained. Therefore, in the following sections, we will focus on the products with the mass ratio of 1:5, and all the referred MIL/CNT–Fe in the following sections are obtained from this condition unless stated otherwise.

The nanostructures of MOF and MIL/CNT–Fe can be further confirmed by transmission electron microscopy (TEM). Figures S6 and 2g show that the yielded spindle-like MIL/CNT–Fe nanorods are threaded with CNTs structures, which have lengths mainly about 200–300 nm and width about 20–40 nm. As proved by the select area electron diffraction (SAED) pattern (Fig. 2f), the (110) plane of metallic Fe is noticed, and the TEM images in Fig. 2g, i show that these Fe nanocrystalline are ranging from 20 to 40 nm. The high-resolution TEM (HRTEM, Fig. 2h) analysis shows that the Fe nanocrystalline exhibits fine crystal structures with a *d*-spacing of 0.201 nm (corresponded to the (110) lattice of metallic Fe). Meanwhile, these Fe nanocrystalline are mainly encapsulated by multilayer graphitic carbon structures with a *d*-spacing of 0.350 nm [39]. These graphitic carbon layers are supposed to be generated by the in situ self-catalysis of Fe nanocrystalline during carbonization, which may benefit the electron transfer and enhance the catalytic activity of MIL/CNT–Fe [21, 40]. Besides, some defects and edge structures can also be observed along the CNTs, thus indicating the catalytic Fe–N_*x*_/pyridinic-N active centers can be preserved to guarantee their good catalytic activities [41].

Based on the recent findings in the literature, the carbonization of Fe-contained MOF with *N*-molecules will allow the abundant doping of Fe–N_*x*_ active sites in the carbon matrix [26, 42–44], which give the catalysts significantly enhanced activities toward several kinds of reactions. The energy-dispersive X-ray spectroscopy (EDX) mapping has been conducted to further research the spatial distribution of elements on the MIL/CNT–Fe-800, and it has been found that the C, N, and Fe elements are uniformly distributed within both the CNTs (Fig. 2j) and the carbonaceous nanorods (Fig. S7), thus indicating the formation of Fe-N_x_ structures [45, 46]. Additionally, the MIL-Fe-800 has also been observed by TEM (Fig. S8), and the MOF shape is maintained, but no CNTs nanostructure can be noticed; meanwhile, many large Fe nanoparticles (ranged from 30 to 180 nm) embedded in the carbon matrix are noticed. Based on earlier findings, the aggregations of big Fe nanoparticles will decrease the catalytic activities of carbon catalysts [40, 44, 47, 48]. The morphology analysis of MIL-Fe-800 and MIL/CNT–Fe-800 reveals that the *N*-molecule plays a vital role in the self-catalyzed carbonization process and the in situ formation of CNT–Fe structures.

**Fig. 3:**
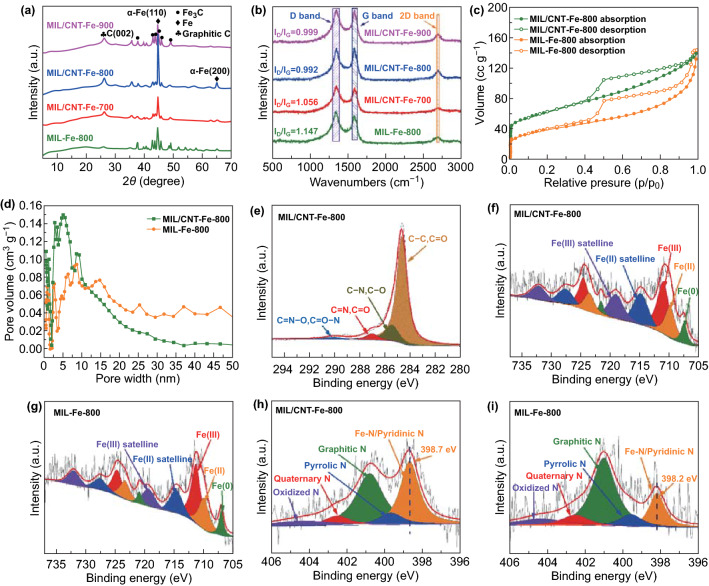
**a** PXRD analysis of different samples. **b** Raman spectra of different samples. **c** N_2_ adsorption–desorption isotherms of catalysts. **d** The pore size distributions of MIL/CNT–Fe-800 and MIL-Fe-800 calculated from the adsorption branch of the isotherms by the quenched solid density functional theory (QSDFT) model for spherical pore type. High-resolution **e** C 1*s*, **f** Fe 2*p*, **h** N 1*s*, XPS spectra of MIL/CNT–Fe-800. High-resolution **g** Fe 2*p* and **i** N 1*s* XPS spectra of MIL-Fe-800

To further confirm the detailed crystal structures of the carbonized products, the powder X-ray diffraction (PXRD) patterns are presented in Fig. 3a, and all samples show sharp peaks at 44.7° and 65.0°, which can be assigned to (110) and (200) planes of metallic Fe nanocrystalline according to the diffraction pattern of α-Fe (JCPDS No. 87-0722). It has been noticed that the MIL/CNT–Fe-800 shows the highest and most intensive peaks of α-Fe and weak peaks of Fe_3_C, thus demonstrating the high crystallinity of metallic Fe, and the formation of Fe_3_C is inhibited due to the self-catalyzed carbonization process, which is consistent with HRTEM and SAED patterns data [49]. For both MIL/CNT–Fe-900 and MIL-Fe-800, the peaks of Fe_3_C are distinct while the peaks of α-Fe are relatively weak, indicating that the metallic Fe has been transferred to Fe_3_C dominantly during pyrolysis due to the high temperature or the absence of *N*-molecule. Meanwhile, the results also suggest that the α-Fe nanocrystalline plays a much more important role than the Fe_3_C, which is consistent with the results of Fenton-like reactions shown in the following sections. After that, the domination of graphitic carbon has been measured by Raman spectroscopy (Fig. 3b). All the carbon hybrids display two intensive peaks around 1350 and 1580 cm^−1^, which can be attributed to the *D* and *G* band, respectively [50]. It is interesting to find that both the intensities of *G* band and 2D band of the MIL/CNT–Fe-800 are distinctly sharper than that of MIL-Fe-800, which indicates for higher graphitic structures due to the *N*-molecule-assisted self-catalyzed carbonization. The similar *D* band intensities indicate that these defects and the porosity of MIL/CNT–Fe-800 can be maintained during carbonization with the etching of NH_3_ gas.

The specific surface areas of MIL/CNT–Fe-800 and MIL-Fe-800 were detected by N_2_ adsorption/desorption measurements [51]. As shown in Fig. 3c, the presence of the hysteresis loop at relative pressures between 0.4 and 0.7 reveals that MIL/CNT–Fe-800 and MIL-Fe-800 possess many micro- and mesopores [32]. MIL/CNT–Fe-800 exhibits uniform mesopores with a dominant size under 10 nm due to the internal pores of CNTs (Fig. 3d). In addition, the calculated Brunauer–Emmett–Teller (BET) surface area has been enhanced from 144 (for MIL-Fe-800) to 222 m^2^ g^−1^ (for MIL/CNT–Fe-800). The microporous surface area and external surface area of the carbon hybrids were analyzed by the *t*-plot method and presented in Table 1. The microporous surface area and total pore volume of MIL/CNT–Fe-800 are also much higher than MIL-Fe-800, which can be attributed to the synergic effects of the CNTs formation and etching of NH_3_ gas to form microporous structures [52].

**Table 1:** Surface area, microporous surface areas, external surface areas, and total pore volume from the BET tests

Samples	BET surface area (m^2^ g^−1^)	^a^Micro-surface area (m^2^ g^−1^)	^a^External surface area (m^2^ g^−1^)	^b^Total pore volume (cc g^−1^)
MIL-Fe-800	144	37	107	0.22
MIL/CNT–Fe-800	222	66	154	0.30

^a^The microporous surface area and external surface area were calculated from the t-plot method with P/P_0_ from 0.4 to 0.6

^b^The total pore volume was calculated from the adsorbed volume at the P/P_0_ of ~ 0.990

To identify the detailed chemical position and electronic structure of catalysts, X-ray photoelectron spectroscopy (XPS) was performed. The XPS survey scanning of MIL/CNT–Fe-800 shows clear N peak, which is much higher than that of MIL-Fe-800, thus indicating more doping of N contents due to the *N*-molecule-assisted carbonization (Fig. S9). As shown in Figs. 3e and S10, the high-resolution C 1*s* spectra are given with all the characteristic peaks of the N-doped carbon structure, especially the 285.5 eV for C–N/C–O bonds. Figure 3f, g shows the curve-fitted high-resolution XPS Fe 2*p* spectra, and the iron should be attributed to Fe^0^/Fe^2+^/Fe^3+^ species. The Fe^0^ valence state confirms the existence of Fe nanocrystalline. The Fe^2+^/Fe^3+^ peaks at 709.8 and 711.3 eV indicate that ionic Fe may bond with N to form Fe–N_*x*_ active sites. Then, as shown in Fig. 3h, i, the high-resolution N 1*s* spectra exhibit that the pyridinic-N in MIL-Fe-800 (398.2 eV) has relatively low binding energy, while this peak measured in MIL/CNT–Fe-800 shifts to 398.7 eV (Fig. 2h), which may be caused by the bonding of Fe^2+^/Fe^3+^ species. Meanwhile, it is found that the other peak positions of N 1*s* species are the same for MIL-Fe-800 and MIL/CNT–Fe-800. Therefore, based on the earlier findings, it is believed that the Fe^2+^/Fe^3+^ species are majorly bonded with pyridinic-N to form the catalytic active Fe–N_*x*_ sites [26]. Notably, as shown in Table 2, the MIL/CNT–Fe-800 exhibits a dramatically increased Fe-N/pyridinic-N contents and significantly decreased graphitic N when compared with MIL-Fe-800. Combining complimentary high-resolution TEM, XRD, Raman, BET, and XPS data, and we are convinced that there are much more Fe/Fe-N_*x*_/pyridinic-N active species, active defects, and porous structures, which may remarkably enhance the catalytic activities of MIL/CNT–Fe-800 in Fenton-like reactions.

**Table 2:** Contents of different N1*s* species in the prepared catalysts

Samples	Fe–N/Pyridinic-N% (398.2 eV)	Fe–N/Pyridinic-N% (398.8 eV)	Pyrrolic N% (399.7 eV)	Graphitic N% (400.9 eV)	Quaternary N% (402.5 eV)	Oxidized N% (404.2 eV)
MIL/CNT–Fe-800	–	35.60	7.93	38.54	8.50	9.41
MIL-Fe-800	17.28	–	9.52	53.47	10.20	9.51

### Fenton-Like Catalytic Degradation of Organic Pollutants

**Fig. 4:**
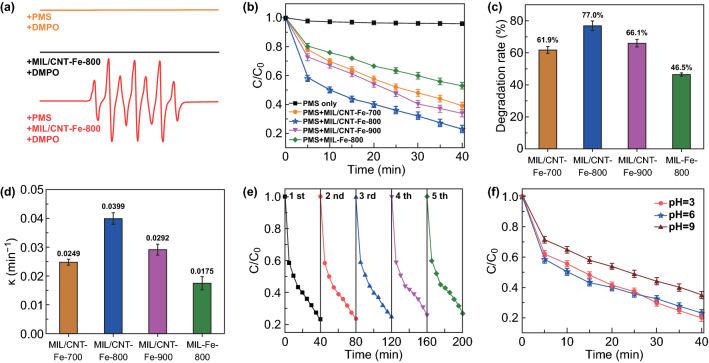
**a** EPR measurements with/without MIL/CNT–Fe-800. **b** Degradation curves of BPA in different reaction systems. **c** Degradation rates (%) of BPA with different catalysts in 40 min. **d** The reaction rate constants of BPA degradation in different reaction systems. **e** Recycling experiments for BPA degradation using MIL/CNT–Fe-800. **f** Contrast experiment at solution with different pH values for BPA degradation using MIL/CNT–Fe-800. Reaction conditions: [BPA] = 20 mg L^−1^, [PMS] = 0.2 g L^−1^, catalyst = 0.1 g L^−1^, T = 298 K, initial pH = 6

The integrated analytical measurements have demonstrated that the *N*-molecule-assisted catalytic carbonization process contributed to the high pore volume and specific surface area, which is stemmed from the CNTs in situ growth and NH_3_ gas etching, thus giving a good balance of graphitization degree and active defects. Meanwhile, the contents of Fe/Fe-N_*x*_/pyridinic-N active species were also improved. These structural advantages imply that the carbon hybrids may possess promising Fenton-like catalytic activities. Herein, to detect the radical generation capabilities during Fenton-like process, the 5,5-dimethyl-1-pyrroline N-oxide (DMPO)-trapped electron paramagnetic resonance (EPR) experiments were performed. As shown in Fig. 4a, without catalyst, no characteristic signal can be detected, and indicating inappreciable radicals is generated by PMS alone. Surprisingly, when PMS and MIL/CNT–Fe-800 are added together, the relative peaks of DMPO present at *a*_N_ = 7.1 G and *a*_H_ = 4.2 G, which can be referenced to the combination between DMPO and singlet oxygen (^1^O_2_) [8]. These analyses give solid evidence for the PMS excitation capability of MIL/CNT–Fe-800 to generate active ^1^O_2_.

To further identify the catalytic performances of the prepared catalyst for the degradation of different types of pollutants, the degradation of BPA (endocrine disruptor) was conducted. As shown in Fig. S11, when considered about the catalytic performance, amount of DCDA, and nanorod structures of MIL/CNT–Fe-800, the mass ratio of MIL-88B-NH_2_: DCDA set at 1:5 shows the optimal condition for the degradation experiments. Then, the carbonization temperature is compared, as shown in Fig. 4b, and all the catalysts show much faster activation of PMS to generate radicals to degrade BPA when compared with PMS alone. Among different carbonization temperatures, the product obtained 800 °C with *N*-molecules, MIL/CNT–Fe-800, shows the best degradation efficiency and as high as 77.0% of BPA can be degraded within 40 min. Compared with MIL/CNT–Fe-700/900, the augmented performance of MIL/CNT–Fe-800 may be attributed to the balanced graphitization degree and active defects. Furthermore, it is found that the MIL-Fe-800 shows the most inferior degradation efficiency (46.5%) for BPA (Fig. 4c).

To further compare the catalytic performances of these carbon catalysts, the BPA degradation kinetics were fitted by the pseudo-first-order reaction. As shown in Fig. 4d, the apparent rate constant of MIL/CNT–Fe-800 has achieved 0.0399 min^−1^, which was more than twice as high as MIL-Fe-800. Meanwhile, the constants of MIL/CNT–Fe-700 and MIL/CNT–Fe-900 can only reach 0.0249 and 0.0292 min^−1^, respectively. Reusability and stability were the critical elements of the application of catalyst; thus, it was imperative to understand the stability of the prepared catalysts. As shown in Fig. 4e, after five recycling experiments, the degradation rate declines slowly, indicating the good stability of MIL/CNT–Fe-800. Besides, in practical application, the pH environment of the pollutants may vary in a large range; thus, the degradation capabilities of these catalysts under different pH conditions have been conducted at different pH = 3, 6, 9. As shown in Fig. 4f, there is a negligible change of degradation efficiency between pH = 3 and pH = 6, when the pH increases to 9, there is a slight decline in degradation rate. Besides, the SEM images of this catalyst after reaction at different conditions are shown in Fig. S12. Overall, the results indicate that the MIL/CNT–Fe-800 can be applied to degrade BPA in wastewater under acidic, neutral, and alkaline conditions.

**Fig. 5:**
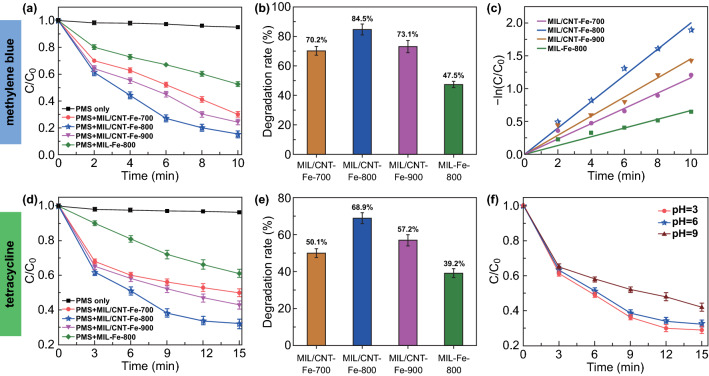
**a** The degradation of MB in different reaction systems. **b** Degradation rates (%) of MB with different catalysts in 40 min. **c** Kinetic linear fitting for the removal of MB, **d** The degradation of TC in different reaction systems. **e** Degradation rate (%) of TC with different catalysts in 15 min. **f** Contrast experiment of different pH for TC degradation using MIL/CNT–Fe-800. Reaction conditions: [MB or TC] = 20 mg L^−1^, [PMS] = 0.2 g L^−1^, catalyst = 0.1 g L^−1^, T = 298 K, initial pH = 6

MB as a typical dye was also applied to test the catalytic degradability of the carbon hybrids. As shown in Fig. 5a, b, the presence of catalysts in PMS solution can lead to high degradation efficiency of MB, which reaches to 70.2%, 84.5%, and 73.1% in 10 min, respectively. The fitting curves of degradation of MB by pseudo-first-order kinetics are shown in Fig. 5c. The highest slope is observed when MIL/CNT–Fe-800 is employed as the catalyst, testifying the highest rate constant of 0.200. Figure S13 shows the influence of pH on the degradation of MB; the results suggest that the catalytic degradation efficiency of MB in acidic and neutral condition is similar, while slightly decreased in alkaline condition as that of the BPA degradation. TC, a frequently used antibiotic, is hardly degraded in the natural environment, which has also been employed as a target to explore the application potential of our catalysts. As shown in Fig. 5d, the MIL/CNT–Fe-800 still exhibits the highest activity for the catalytic degradation of TC and exhibits the fastest degradation rate (68.9% of TC degradation within only 15 min, Fig. 5e). The influence of pH on degradation of TC by employing MIL/CNT–Fe-800 shows similar variations with the degradation of BPA and MB (Fig. 5f).

Altogether, the above measurements determined the dramatical promotion of catalytic degradation performance of MIL/CNT–Fe-800, which is much superior to the control sample, MIL-Fe-800. It is believed that the surprising Fenton-like catalytic degradation efficiency of MIL/CNT–Fe-800 toward pollutants is benefited from the *N*-molecule-assisted catalytic carbonization, which gives the catalysts with enriched Fe/Fe-N_*x*_/pyridinic-N active species, abundant micro-/meso-porous structures, and balanced graphitization degree and active defects.

**Fig. 6:**
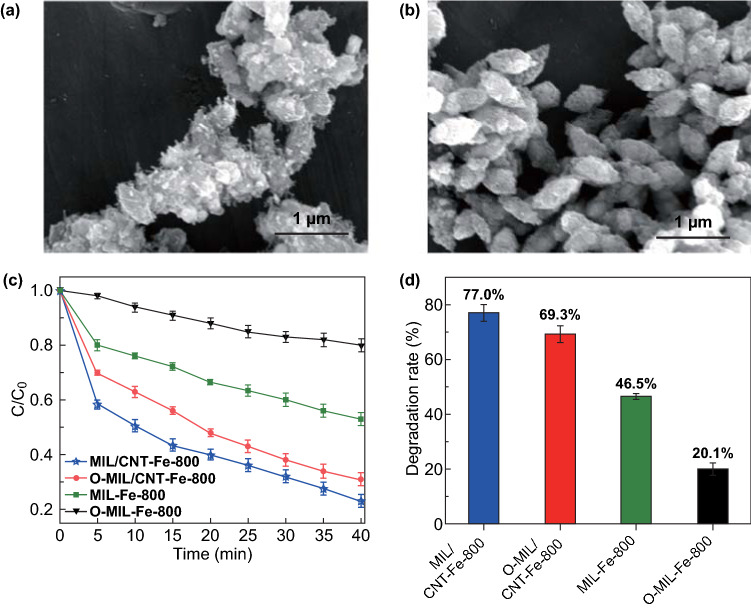
**a** SEM image of O-MIL/CNT–Fe-800. **b** SEM image of O-MIL-Fe-800. **c** Contrast experiment of iron oxide for BPA degradation. **d** Degradation rate (%) of BPA with different catalysts in 40 min

Based on the current findings, the catalytic activities of metal-N_x_ and pyridinic-N active species are very high for Fenton-like reactions [8, 53]. The catalytic performance of Fe nanocrystalline and Fe_2_O_3_ has not been clearly compared [46]. In this study, we also want to confirm the catalytic activities of Fe further are more active than its Fe_2_O_3_ form in the degradation of pollutants. First, the MIL/CNT–Fe-800 and MIL-Fe-800 are oxidized at 350 °C to obtain the O-MIL/CNT–Fe-800 and O-MIL-Fe-800 with abundant Fe_2_O_3_, which has been demonstrated by the PXRD patterns (Fig. S14) [54]. As shown in Fig. 6a, b, the previous structures of MIL/CNT–Fe-800 and MIL-Fe-800 are almost preserved. For the catalytic degradation capabilities, the performance of O-MIL/CNT–Fe-800 and O-MIL-Fe-800 both declines greatly after oxidation as shown in Fig. 6c, d. The degradation rate in 40 min of MIL/CNT–Fe-800 and MIL-Fe-800 decreases from 77.0 to 69.3% and 46.5 to 20.1%, respectively. Though much more consistent and comparable conditions are needed to give a convincing result, our results give a preliminary data to suggest that the catalytic performance of Fe nanocrystalline may be higher than Fe_2_O_3_ in the carbon material-based Fenton-like catalysis due to its better conductivity and high intrinsic catalytic centers.

## Conclusion

In summary, we report a facile *N*-molecule-assisted self-catalytic carbonization process that augments the intrinsic Fenton-like activity of MOF-derived carbon materials for the degradation of different pollutants in wastewater. During the pyrolysis, the DCDA as *N*-molecules supplies alkane/ammonia gases and the formed iron nanoparticles act as catalysts, which facilitate the elaborated formation of CNTs and micro-/meso-porous structures via the process of chemical vapor deposition and ammonia gas etching. Furthermore, this unique carbonization process significantly augments its Fenton-like activities due to the synergic effects of the increased Fe/Fe-N_*x*_/pyridinic-N active species, porous structures, and conductive CNTs structures. Consequently, these carbon hybrids exhibit high removal efficiency of endocrine disruptor (BPA), industrial dye (MB), and widely used antibiotic in cultivation industry (TC). Overall, this work not only provides a viable pathway for fabricating MOF-derived nanomaterials with rationally designed structures and high intrinsic Fenton-like activities but also creates many opportunities in the future design of advanced carbon hybrids for efficient energy and environmental applications.

## Electronic supplementary material

Below is the link to the electronic supplementary material.

Supplementary material 1 (PDF 998 kb)
