# Supplementary material for: Augmenting Intrinsic Fenton-Like Activities of MOF-Derived Catalysts via N-Molecule-Assisted Self-catalyzed Carbonization
Source: Nanomicro Lett. 2019 Oct 17;11:87. doi: 10.1007/s40820-019-0319-4 (PMC7770684; doi:10.1007/s40820-019-0319-4)
Supplement: Supplementary file 1 — Supplementary material 1 (PDF 998 kb) [file 40820_2019_319_MOESM1_ESM.pdf]

Supporting Information for

Augmenting Intrinsic Fenton-like Activities of MOF-derived Catalysts via N-Molecules Assisted Self-catalyzed Carbonization

Chengdong Yang[1], Mi Zhou[1], Chao He[2], Yun Gao[1], Shuang Li[3], Xin Fan[1], Yi Lin[1, *], Fei Cheng[1], Puxin Zhu[1, *], Chong Cheng[2, 4]

[1]Textile Institute, College of Biomass Science and Engineering, Sichuan University, Chengdu 610065, People's Republic of China

[2]College of Polymer Science and Engineering, State Key Laboratory of Polymer Materials Engineering, Sichuan University, Chengdu 610065, People's Republic of China

[3]Functional Materials, Department of Chemistry, Technische Universität Berlin, Hardenbergstraße 40, 10623 Berlin, Germany

[4]Department of Chemistry and Biochemistry, Freie Universität Berlin, Takustrasse 3, 14195 Berlin, Germany

*Corresponding authors. E-mail: linasdn@126.com (Y. Lin); zhupxscu@163.com (P. Zhu)

S1 Characterization of Catalyst

The powder X-ray diffraction (PWXD) was performed by Philips X' Pert Pro MPD system with Cu radiation at a voltage of 40 kV. The sample was scanned in a scanning 2θ range from 5° to 80°. The surface morphology of catalyst was observed by field emission scan electron microscope (FE-SEM, Apreo S HiVac FEI). The high-resolution transmission electron microscope (HR-TEM) was conducted by Tecnai G2 F20 S-TWIN operated at 200 kV. The chemical structures of catalysts were measured by X-ray photoelectron spectroscopy (XPS), which was recorded on the ESCAL 250 XPS equipped with a monochromatic Al K-α source. The electron paramagnetic resonance (Bruker EPR EMX Plus) spectra were conducted on with a center field at 3500 G, and a sweep width of 140 G. N_2 sorption analysis was conducted on ASAP 2460 Micromeritics. The surface area was calculated using the Brunauer-Emmett-Teller (BET) calculation. The pore size distribution (PSD) plot was recorded from the adsorption branch of the isotherm based on the QSDFT model for spherical pore.

S2 Supplementary Figures

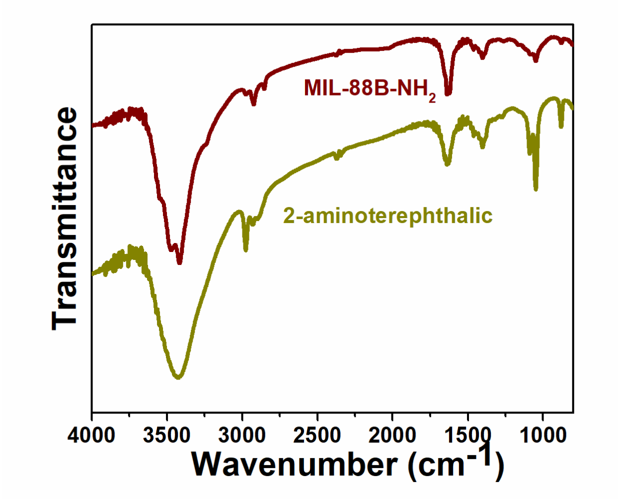

Fig. S1 FTIR spectra of MIL-88B-NH_2 and 2-aminoterephthalic

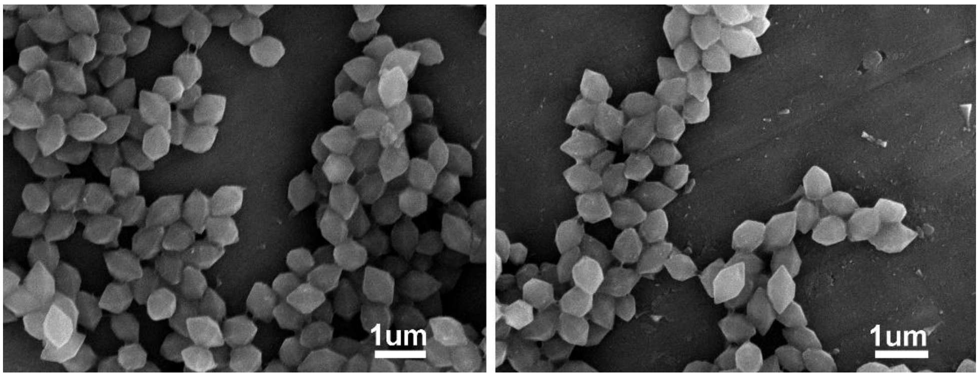

Fig. S2 SEM images of surface morphology of MIL-88B-NH_2

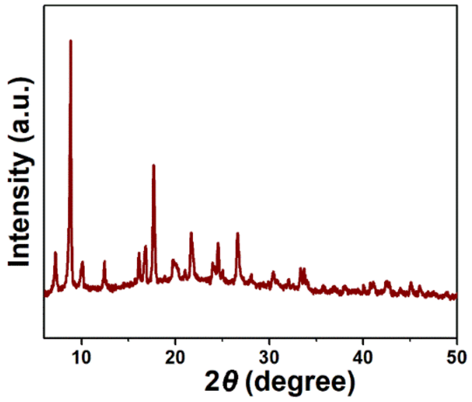

Fig. S3 PWRD spectrum of MIL-88B-NH_2 before pyrolysis

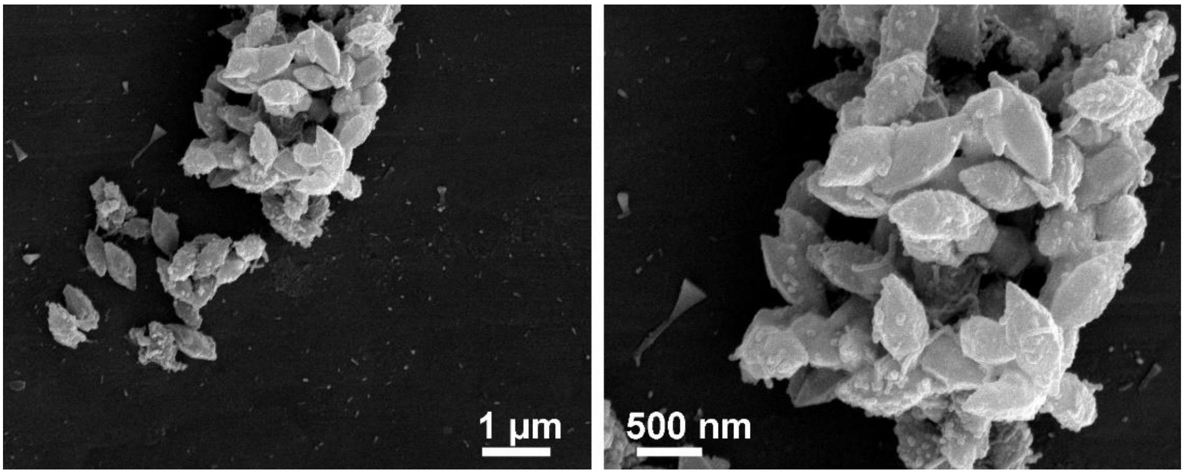

Fig. S4 SEM images of MIL/CNT-Fe-3

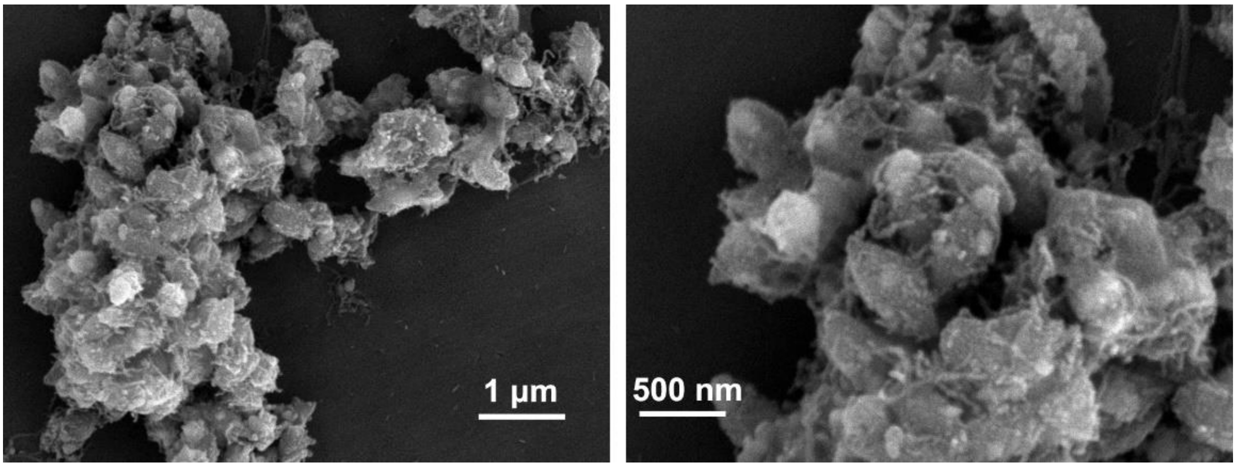

Fig. S5 SEM images of MIL/CNT-Fe-8

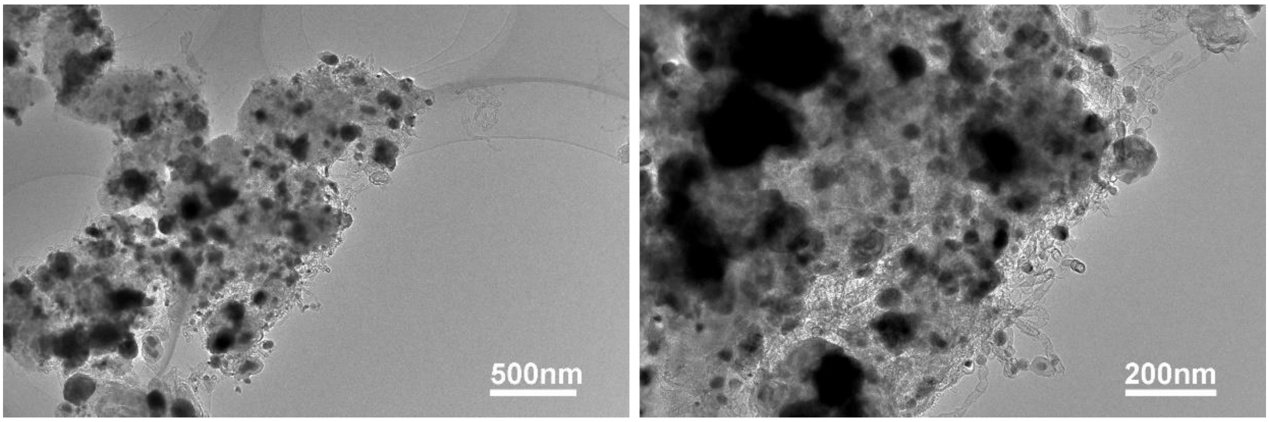

Fig. S6 TEM images of MIL/CNT-Fe-800

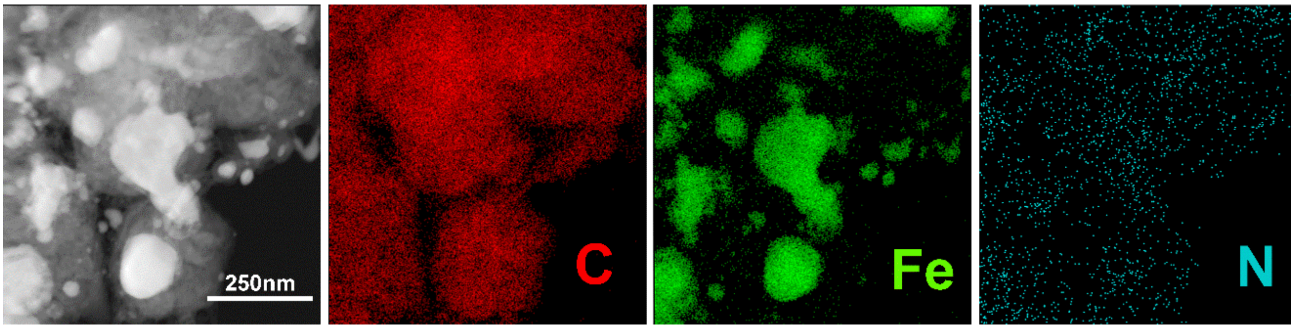

Fig. S7 EDX mapping images of MIL/CNT-Fe-800

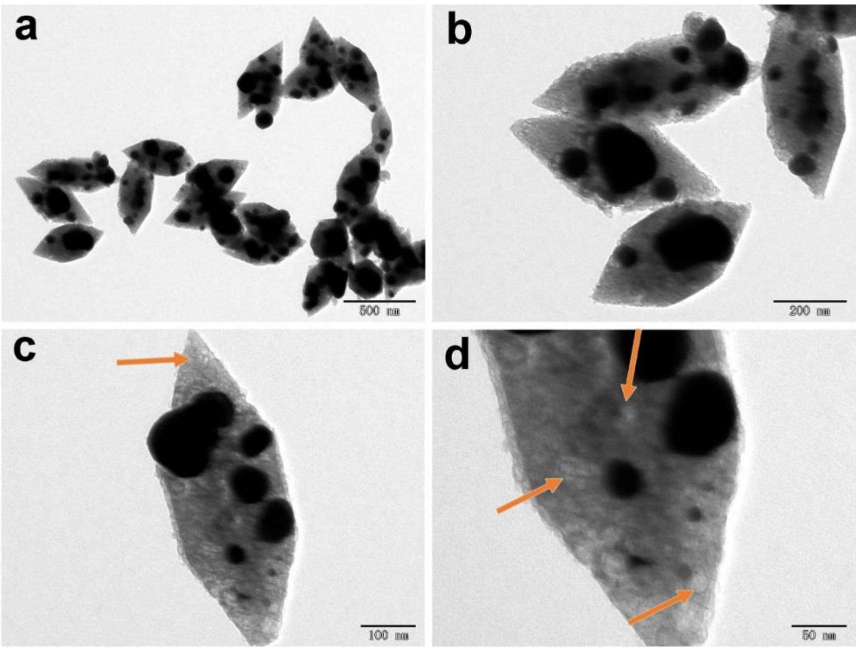

Fig. S8 The TEM images of MIL-Fe-800 at different magnifications. Mesoporous are marked with yellow arrows

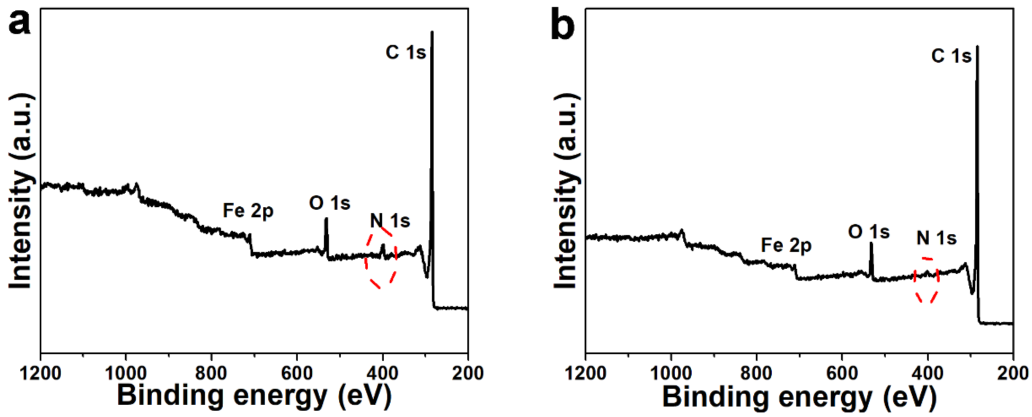

Fig. S9 XPS survey scanning of **a** MIL/CNT-Fe-800 and **b** MIL-Fe-800

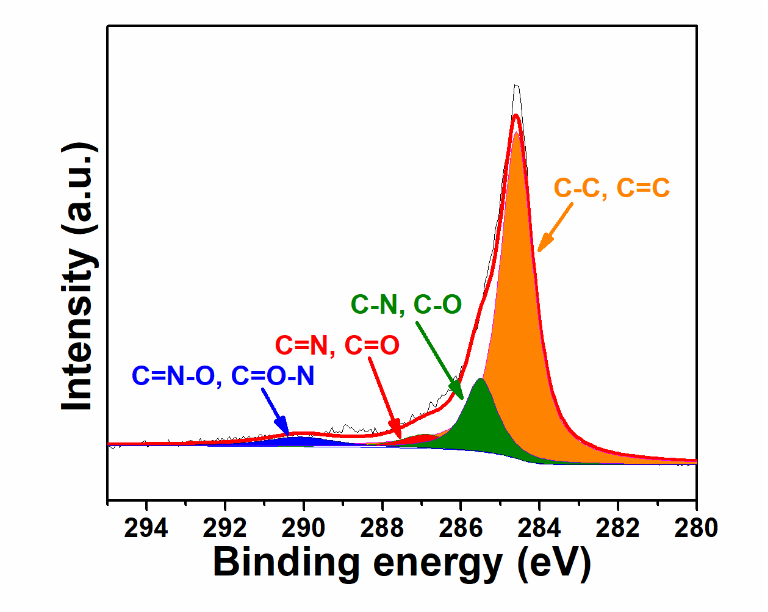

Fig. S10 The deconvolution of magnified C1s XPS spectra for MIL-Fe-800

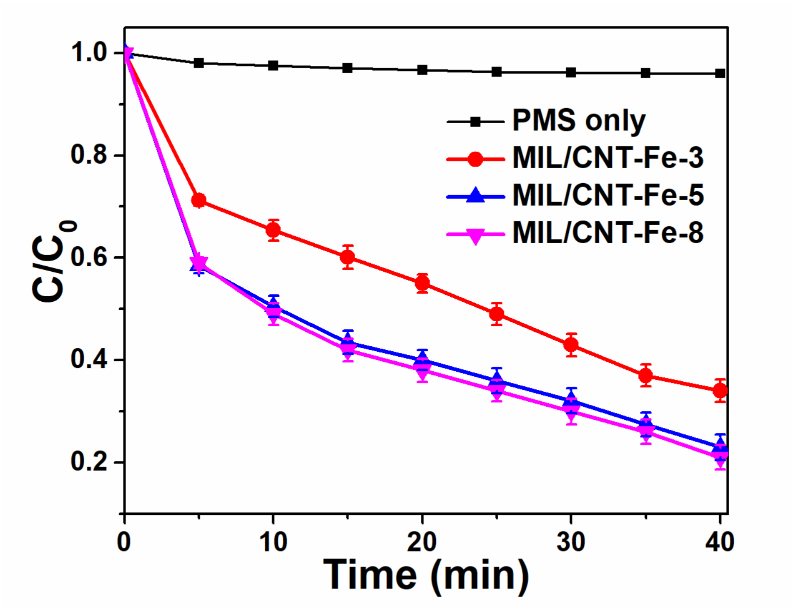

Fig. S11 The degradation of BPA in different catalysts synthesized from different mass ratios of N-molecules

The results show that both the MIL/CNT-Fe-5 and MIL/CNT-Fe-8 show higher catalytic performance than MIL/CNT-Fe-3 in the degradation of BPA. Considered about the similar performance, higher amount of DCDA, and collapse of nanorod structures of MIL/CNT-Fe-8 (Fig. S5). The MIL/CNT-Fe-5 with a mass ratio of MIL-88B-NH_2:DCDA set at 1:5 is the best selection for the degradation experiments.

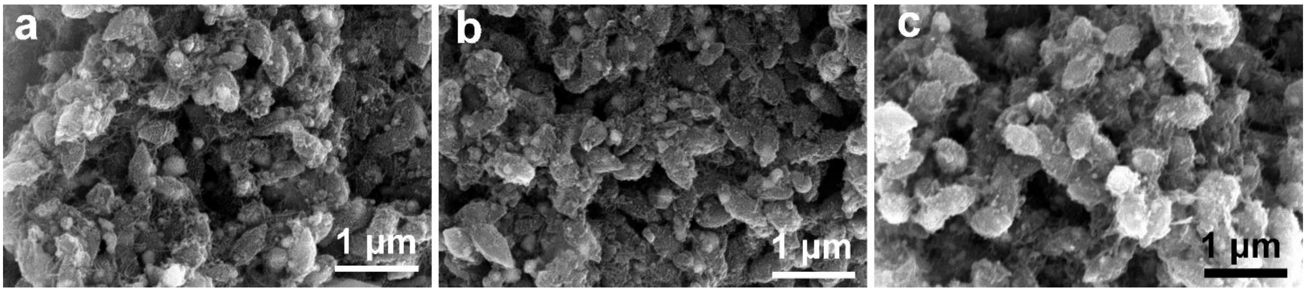

Fig. S12 The SEM images of MIL/CNT-Fe-800 after the degradation of BPA at **a** acidic,**b** neutral, and **c** alkaline conditions

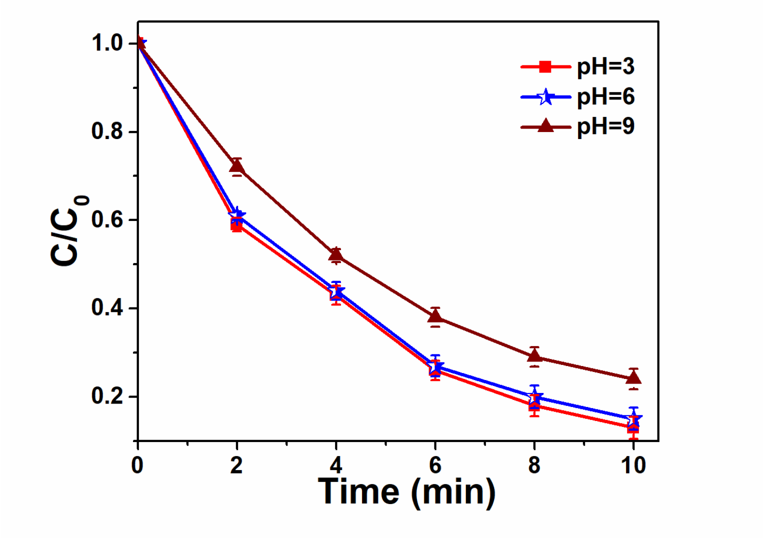

Fig. S13 Contrast experiment of different pH for MB degradation using MIL/CNT-Fe-800

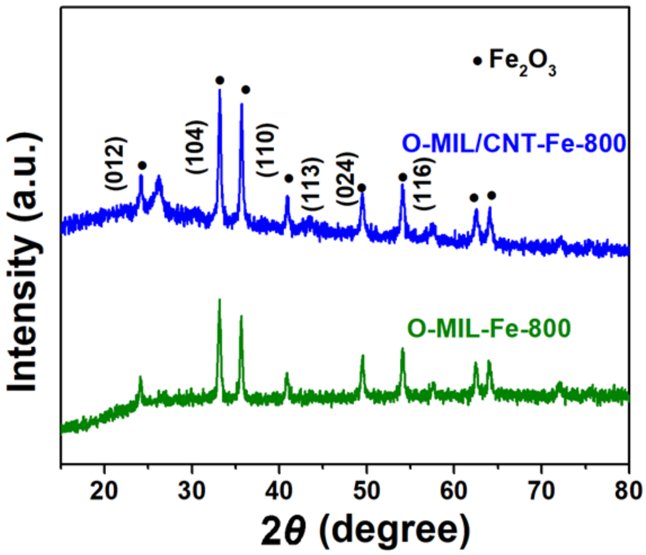

Fig. S14 PXRD spectra of O-MIL/CNT-Fe-800 and O-MIL-Fe-800
